# The Genetics of Monogenic Frontotemporal Dementia

**DOI:** 10.1590/1980-57642015DN93000003

**Published:** 2015

**Authors:** Leonel T. Takada

**Affiliations:** 1 MD, PhD, Cognitive and Behavioral Neurology Unit, Department of Neurology, Hospital das Clínicas da Faculdade de Medicina da Universidade de São Paulo, Brazil.

**Keywords:** frontotemporal lobar degeneration, frontotemporal dementia, genetics, amyotrophic lateral sclerosis

## Abstract

Around 10-15% of patients diagnosed with frontotemporal dementia (FTD) have a positive family history for FTD with an autosomal dominant pattern of inheritance. Since the identification of mutations in *MAPT* (microtubule-associated protein tau gene) in 1998, over 10 other genes have been associated with FTD spectrum disorders, discussed in this review. Along with *MAPT*, mutations in *GRN* (progranulin) and *C9orf72* (chromosome 9 open reading frame 72) are the most commonly identified in FTD cohorts. The association of FTD and motor neuron disease (MND) can be caused by mutations in *C9orf72* and other genes, such as *TARDBP* (TAR DNA-binding protein), *FUS* (fused in sarcoma), *UBQLN2* (ubiquilin 2). Multisystem proteinopathy is a complex phenotype that includes FTD, Paget disease of the bone, inclusion body myopathy and MND, and can be due to mutations in *VCP* (valosing containing protein) and other recently identified genes.

## Abstract (pt)

Cerca de 10-15% dos pacientes diagnosticados com demência frontotemporal (FDFT) têm uma história familiar positiva para DFT com um padrão de herança autossômico dominante. Desde a identificação de mutações em *MAPT* (proteína tau associada a microtúbulos), em 1998, mais de 10 outros genes já foram associados a doenças do espectro da DFT, que são discutidas nesta revisão. Junto com *MAPT*, mutações em *GRN* (progranulina) e *C9orf72* (*chromosome 9 open reading frame 72*) são as mais comumente identificadas em casuísticas de DFT. A associação de DFT com doença do neurónio motor (DNM) pode ser causada por mutações em *C9orf72* e outros genes, tais como *TARDBP* (*TAR DNA-binding protein*), *FUS* (*fused in sarcoma*), *UBQLN2* (ubiquilina 2) e outros genes. Proteinopatia multissistêmica é um fenótipo complexo que inclui DFT, doença de Paget óssea, miopatia com corpúsculos de inclusão e DNM, e pode ser devida a mutações em *VCP* (*valosin containing protein*) e outros genes recentemente identificados.

## INTRODUCTION

The term frontotemporal dementia (FTD) refers to a group of diseases characterzed by focal degeneration of temporal and/or frontal lobes of the brain. Clinically, FTD is subclassified into a behavioral variant (bvFTD) and into two language variants, named agrammatic or nonfluent variant of Primary Progressive Aphasia (nfvPPA) and semantic variant of PPA (svPPA).^1,2^ Progressive supranuclear palsy (PSP) and corticobasal syndrome (CBS) often overlap clinically with FTD spectrum disorders, as behavioral symptoms and/or language impairment similar to those seen in FTD can appear in patients with PSP or CBS, leading to the inclusion of both (by some authors) within the clinical spectrum of FTD.^3^ There is also significant overlap between FTD and motor neuron disease (MND), as around 15% of patients with bvFTD are also diagnosed with amyotrophic lateral sclerosis (ALS).^4^

Whereas FTD is used to characterize clinical syndromes, the term Frontotemporal Lobar Degeneration (FTLD) is used in the neuropathological classification of this group of disorders. FTLD is subclassified according to the main aggregated protein found in neuronal and/or glial inclusions into: FTLD-tau (tau protein), FTLD-TDP (TAR DNA binding protein with 43kDA, or TDP-43) and FTLD-FUS (fused in sarcoma protein, or FUS).^5^ FTLD-TDP represents around 50% of FTLD cases, FTLD-tau around 45%, while FTLD-FUS is observed in 5-10% of FTLD cases. The main protein has not been identified in a small subset of cases, and these are diagnosed as FTLD-U, since the inclusions are immunoreactive to antibodies against proteins from the ubiquitin-proteasome system, but not to tau, TDP-43 or FUS.^5^ Again, overlap with PSP, CBS and MND is seen, as FTLD-tau might underlie PSP and CBS, and inclusions with TDP-43 and FUS can be found in MND.

FTD has an important genetic component, as around 40% of patients have at least one first-degree relative with an FTD spectrum disease, and about 10-15% have a family history of FTD suggesting an autosomal dominant pattern of inheritance.^6^

Since identification of mutations in the gene of microtubule-associated protein tau (*MAPT*) as a cause of monogenic FTD in 1998,7 mutations in over ten other genes have been identified, such as in the progranulin gene (*GRN*) in 2006, and in *C9orf72* (Chromosome 9 open reading frame 72) in 2011.8-11 Mutations in *MAPT*, *GRN* and *C9orf72* are the most commonly reported in FTD cohorts worldwide.

**Figure 1:**
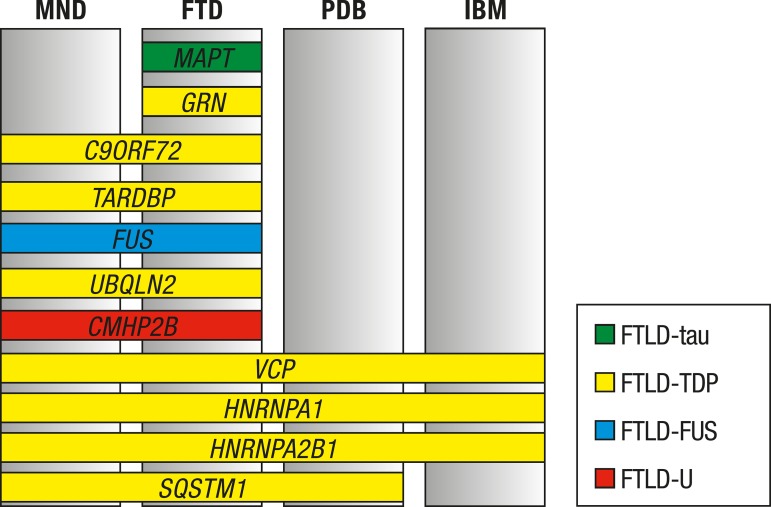
Genes associated with monogenic FTD with clinical and neuropathological correlations

MND: motor neuron disease; FTD: frontotemporal dementia; PDB: Paget disease ofthe bone; IBM: inclusion body myositis. Each color refers to a FTLD subtype. Please see text for more information regarding neuropathology. Even though TDP-43 pathology is present in *UBQLN2* mutations, inclusions immunostained by antibodies against ubiquilin 2 have also been reported, and ubiquilin 2 might be the major abnormal protein in this mutation. Mutations in *HNRNP1* and *HNRNPA2B1* are still lacking neuropathological studies confirming TDP-43 pathology in the central nervous system.

Mutations in *MAPT* and *GRN* typically present as FTD phenotypes, whereas hexanucleotide repeat expansions in *C9orf72* are associated with FTD and MND (Figure 1). Mutations in other genes, such as in those encoding TDP-43 (*TARDBP*) or FUS (*FUS*) also present clinically as FTD and/or MND.^12^

FTD can also be part of a complex phenotype called multisystem proteinopathy (MSP) that can be caused by single mutations, such as in *VCP*. MSP is a rare syndrome characterized by a combination of the following phenotypes: bvFTD, MND, Paget disease of the bone and/or inclusion body myopathy.^13^

## GENES ASSOCIATED WITH MONOGENIC FORMS OF FTD

***MAPT* GENE.** The *MAPT* gene is located on chromosome 17q21.1. It encodes the microtubule-associated protein tau, which stabilizes and promotes the assembly of microtubules.^14^

There are six isoforms of tau in the normal brain, generated from the alternative splicing of exons 2, 3 and 10.14 Exons 9-12 encode the microtubule-binding domains and, depending on the inclusion or exclusion of exon 10 from the transcripts, the isoforms may have three (3R) or four repetitions (4R) of these domains. In normal brain, 3R and 4R isoforms coexist in equal proportions (1:1), and changes in this ratio are believed to lead to neurodegeneration.^7,14^

*MAPT* mutations can be divided into two groups, with different pathogenic mechanisms.^7,14^ The first group, composed of missense mutations and deletions, modifies tau protein and its function in order to increase or decrease its interaction with microtubules. Some of these mutations may lead to a greater tendency to form tau filaments that form toxic aggregates. The second group of mutations (located in exon 10 or flanking regions) interfere with the alternative splicing of exon 10, changing the 3R:4R tau ratio (increasing 4R tau), which leads to an increase in filamentous inclusions and is associated with neurodegeneration.^14^

More than 44 pathogenic mutations (missense, deletions, silent or intronic mutations) have been reported in the literature.^15^ The pathogenic gene mutations are clustered in exons 9-13, with the exception of two mutations in exon 1.^15^

In FTD cohorts with more than 100 patients, *MAPT* mutations were found in 3-11% of cases (9-38% among familial FTD cases, and 0-3% among sporadic FTD cases).^9,12,16-21^ *MAPT* mutations cause disease with autosomal dominant inheritance and penetrance greater than 95%.^22^

The mean age of onset of symptoms varies between 46 and 57 years, and the age at onset ranges between 17 and 75 years.^12,16^ Onset after 70 years of age is rare.^12^ The duration of the disease until death is also variable (on average 8 to 10 years), but cases with duration of disease ranging from one to 25 years have been described.^12,16,17^

BvFTD, with or without Parkinsonism, is the most commonly reported phenotype.^12,16^ PSP^12^ and svPPA^17^ are also relatively frequent, whereas CBS, nfvPPA^12^ and early-onset dementia of the Alzheimer type^23^ are less frequently observed. The Alzheimer's disease (AD) phenotype has been described in *MAPT* mutations, but the neuropathological diagnosis was not AD in any of the cases reported.^23^

Parkinsonism is reported in 25-56% of cases, and oculomotor abnormalities in 13-29%.^12,16,24^ Oculomotor changes appear to differentiate *MAPT* mutations from *GRN* or *C9orf72* mutations, as they are infrequent in the latter.^12,16^

The presence of DNM is very rare in *MAPT* mutations. Recently, a new mutation (p.D348G) was found, a kindred with lower MND and respiratory failure, but without signs of cognitive impairment.^25^ Progressive hypoventilation has also been reported in the only family described to date with a homozygous *MAPT* mutation (p.S352L) although in the homozygous mutation, symptom onset occurred much earlier and both siblings died before five years of age.^26^

Structural neuroimaging studies have shown that the atrophy is more significant in anteromedial temporal regions in patients with symptomatic *MAPT* mutations.^27^ Atrophy is also observed in the frontal lobes, particularly in the orbitofrontal regions, and is typically symmetric (contrasting with the asymmetry observed in *GRN* mutations).^27^

The neuropathological changes described in *MAPT* mutations are heterogeneous, but typically neuronal or glial, and neuronal inclusions with hyperphosphorylated tau are observed.^28^ Mutations located in exons 9, 11, 12 and 13 are generally associated with inclusions in neurons that contain predominantly 3R tau, whereas those located in exons 1 and 10 or in introns flanking exons 9 and 10 tend to generate inclusions in neurons and glial cells in which 4R is predominant.^28^

***GRN* GENE.** The progranulin gene is located in the 17q21.32 region and is composed of 13 exons. The gene encodes progranulin, a protein with 593 amino acids which can be cleaved into granulin peptides (A, B, C, D, E, F, and P).^29^ Progranulin is mainly expressed in epithelial and hematopoietic cells; and is expressed in neurons and microglia in the nervous system. The protein has properties as a neurotrophic growth factor and in neuronal survival (protecting neurons from toxic insults in laboratory models). Furthermore, it participates in inflammatory processes (such as wound healing, microglia activation and inhibition of TNF-α), tumorigenesis, and is increased in diabetes mellitus.^29^

Pathogenic mutations in *GRN* lead to loss of function, and cause disease through haploinsufficiency.^8^ Null mutations in *GRN* are most frequently nonsense, or insertions or deletions that generate a frameshift mutation with premature stop codon formation and subsequent nonsense-mediated mRNA decay. Mutations located in splicing or translation initiation sites, and (partial and total) gene deletions have also been reported.^15,29^ Missense mutations have also been described, but there is evidence of pathogenicity for only a few of these - affecting the production, secretion or function of progranulin - demonstrated by functional studies, including: p.A9D, p.C139R, p.R432C and p.C521Y.^30^

Experiments done in animal and cell culture models have shown that progranulin deficiency causes reduction in synaptic density, decreased survival of neurons, increased susceptibility of neurons to stressors, and abnormally increased inflammatory responses, which might be the mechanisms underlying neurodegeneration.^29^

Over 70 mutations have been reported in the literature.^15^ In FTD cohorts with more than 100 patients, mutations in *GRN* were found in 4-12% of cases (7-28% among familial and 1-4% among sporadic FTD cases).^12,16-20,31^ Mutations have an autosomal dominant pattern of inheritance, with an estimated penetrance in 50-60% at 60 years and greater than 90% at age 70.^20,31,32^

The mean age of onset of symptoms ranges from 57 to 62 years, and age at onset ranges from 35 to 87 years,^12,16-20,31^ Besides the phenotypic variability among families, there is great variability in the clinical presentation among individuals of the same family, with differences in age of onset of symptoms varying by up to 20 years.^32^ The duration of symptoms is on average 5-9 years (range 1-23 years).^12,16,17,19,31^

The most common clinical presentation is bvFTD, followed in order of frequency by nfvPPA and CBS.^12,16,19,31^ Mutations in *GRN* were the most frequent cause of familial CBS in some series.^12,31^ Other PPA variants, such as semantic variant and mixed variant, have been reported in a few cases.^19^ Alzheimer-type dementia, Parkinson's disease and dementia with Lewy bodies are infrequent phenotypes; and PSP is rare.^31,33,34^ The bvFTD with MND presentation is also very rare and has been reported only in a few isolated cases.^20,31^

Parkinsonism is observed in 20-50% of patients.^12,16,32^ One group reported an increased frequency of visual hallucinations (around 25%) in individuals with *GRN* mutations,^12^ but other groups failed to confirm the same finding, and found frequencies of hallucinations and/or psychosis of between 5 and 12%.^19,32^ In addition to executive dysfunction signs (typical of bvFTD), neuropsychological assessment may show signs of parietal lobe dysfunction, such as dysgraphia, dyscalculia, apraxia and/or visuospatial deficits.^19^

A homozygous *GRN* mutation was reported in two siblings with adult-onset neuronal ceroid lipofuscinosis, showing that homozygous mutations in *GRN* lead to a completely different phenotype of heterozygous mutations. Neuronal ceroid lipofuscinosis is a lysosomal storage disease, and this finding suggests that progranulin participates in cellular processes related to lysossomes.^35^

Structural neuroimaging studies indicate that the asymmetry is more significant in *GRN* than in *MAPT* or *C9orf72* mutations.^19,36^ Atrophy is more prominent in posterior temporal and parietal regions, but also extends to the frontal lobes.^27^ Parietal regions are significantly more atrophied in *GRN* than in *MAPT* mutations.

Periventricular subcortical hyperintensities indicating involvement of the white matter on T2-weighted and FLAIR MRI images were observed in 20-40% of patients, and are found mainly in regions of maximal atrophy such as the frontal and parietal lobes.^37,38^ The hyperintensities can be confluent, and affect U-fibers as well as subcortical white matter.^37^ The neuropathological substrates of these white matter changes remain unknown, but are possibly related to demyelination and/or microglia activation.^38^

*GRN* null mutations lead to reductions in the levels of progranulin in plasma and cerebrospinal fluid, both in symptomatic and pre-symptomatic individuals.^39^ Missense mutations may also be associated with reduced levels of progranulin, similar to those of null mutations, with intermediate values ​​between those found in null mutations and controls, or even with normal levels, which would be a limiting factor for the use of plasma progranulin measurement as a screening method for *GRN* mutations.^39^

Mutations in *GRN* lead to accumulation of intracellular inclusions with hyperphosphorylated TDP-43 and, in the neuropathological study of brains of individuals with *GRN* mutations, the pattern of TDP-43 inclusions is consistent with FTLD-TDP type A.^40^ In FTLD-TDP type A, neuronal cytoplasmic inclusions immunoreactive to antibodies against TDP-43 are frequent, as are short dystrophic neurites where abnormalities are mainly found in layer 2 of the cortex.^40^ The mechanism by which a deficiency in progranulin leads to neurodegeneration and abnormal TDP-43 accumulation in the brain is not yet known. It is possible that the abnormal accumulation of TDP-43 is related to increased susceptibility to external stressors than nerve cells present in progranulin deficiency, since the TDP-43 protein is involved in cellular processes activated during stress, such as the formation of stress granules, while the solubility and aggregation TDP-43 change during cellular stress.^29^

## *C9ORF72* AND OTHER GENES ASSOCIATED WITH FTD-MND

***C9ORF72* gene.** GGGGCC hexanucleotide repeat expansions in an intronic region of *C9orf72* were discovered in 2011 by two independent groups as a cause of FTD, FTD-MND and ALS.^9,10^

The *C9orf72* gene is located on chromosome 9p21.1. The function of the C9ORF72 protein is still not well understood but there is evidence to suggest it regulates processes related to the endosomal system and autophagy, one of the protein degradation pathways.^41^

The mechanisms by which GGGGCC expansions lead to neurodegeneration remain unclear, but important discoveries have been made. One of the most important findings was that the hexanucleotide repeats form proteins with repeats of dipeptides (polyGA [glycine-alanine], polyGP [glycine-proline], polyGR [arginine-glycine], polyPR [proline-arginine] and polyPA [proline-alanine]) through repeat-associated non-ATG-initiated translation (RAN translation).^42^ Based on the currently available evidence, it is believed that the hexanucleotide repeat expansion in *C9orf72* leads to neurodegeneration either by a dual mechanism, i.e. through RNA- and polypeptide-mediated toxicity, or by toxicity mediated by polypeptides only.^43^

The exact number of repetitions required to cause disease has not been established due to the fact that the currently available technologies for sequencing are not able to sequence thousands of base pairs per read. In general, it is accepted that 30 or more hexanucleotide repeats are pathogenic, although it is estimated that the number of repeats in most patients is around 700-4400.^9,10,44,45^

The inheritance pattern is typically autosomal dominant, but the expansion has also been found in homozygosity, with a similar phenotype to that seen in heterozygous expansions.^46^ The penetrance is high, but is probably incomplete, as the mutation can be found in cognitively normal elderly and it is estimated that 0.1-0.2% of the normal population carries the pathogenic expansion.^44,45^ A multicenter study estimated penetrance of 58% at age 58 years and close to 100% at age 80.45

Diseases caused by repeat expansions can be associated with the anticipation phenomenon. In the case of expansions in *C9orf72*, the study of anticipation is limited by the lack of techniques to accurately measure the number of expansions, as previously mentioned. Another difficulty for studies of anticipation (and penetrance) is that the expansion is unstable, and even monozygotic twins, and different tissue samples from the same individual, might have expansions with different numbers of repeats.^44,47^ In addition, there is evidence that mosaicism is age dependent, which further complicates the interpretation of the number of repeats.^47^ Nonetheless, a few studies have suggested there is anticipation, with the age of onset of symptoms in individuals of a generation occurring on average 7-11 years earlier than in the previous generation.^48,49^

In Europe, the mutation was found in 10% of FTD cases (18% of familial FTD and 6% of sporadic FTD) and in 9% of cases of ALS (37% of familial ALS and 6% of sporadic ALS).^45,50^ In familial FTD-MND, 56% had the mutation, and in sporadic FTD-MND, 14% had the mutation.^50^

The most commonly observed phenotypes are bvFTD, FTD-MND and ALS.^9,10^ The three phenotypes can appear in individuals of the same family, but within a family only one or two of these phenotypes might also appear. Phenotypic heterogeneity is also observed, with age of onset of symptoms varying by more than 20 years, and duration of disease varying by more than 10 years, within a single kindred.^49^

The mean age at symptom onset in bvFTD varies between 49 and 67 years, and age at onset ranges from 18 to 76 years.^12,21,48,50,51^ Clinically, bvFTD caused by this mutation is generally similar to that of sporadic cases, but its onset can occur slightly earlier.^48^ Additionally, some studies suggest that psychotic symptoms are more common in patients with the mutation than in sporadic cases, particularly in the early stages of disease. The neuropsychological profile is typical of bvFTD, characterized by executive dysfunction, but decline in episodic memory and visuospatial skills were reported in some patients.^21^ Parkinsonism occurs in 12-35% of patients and is less frequent than in *MAPT* or *GRN* mutations.^12,16,18^

PPA is not a common presentation, and represented 0-20% of FTD cases due to *C9orf72* repeat expansions in different cohorts.^12,45,51^ However, both svPPA and nfvPPA have been reported with this mutation, even associated with MND. MND is found in about 30% of cases of bvFTD and usually presents as ALS.^50^ The mean age of onset of ALS is 56.8 years, ranging from 27 to 89, and is similar to sporadic ALS cases.^45^

Aside from FTD, FTD-MND, or MND, other phenotypes are infrequent or rare in this mutation. There have been reports of AD, including cases with neuropathological confirmation in individuals with *C9orf72* repeat expansions, but in most cohorts, the mutation was found in <1% of AD cases.^33,44^ Other phenotypes have been described in a few cases, or as case reports, such as Parkinson's disease (including one with neuropathological confirmation), CBS with or without ALS, PSP with or without ALS, dementia with Lewy bodies, cerebellar ataxia, and ALS with multiple sclerosis.^52-55^ Repeat expansions in *C9orf72* were also found in about 2% of patients diagnosed with Huntington-like syndrome, and this may be the most common mutation in this diagnostic group.^44^

The duration of symptoms is variable, with mean duration reported in different studies of between 3 and 15 years (disease duration ranging from 1 to 24 years).^12,16,18,51^ Onset of symptoms is sometimes difficult to identify accurately, as in some cases, behavioral changes occur slowly years or decades before a dementia diagnosis can be established.^49^ The rate of progression of the disease also varies significantly, and both slowly and rapidly progressive forms of bvFTD caused by mutation in *C9orf72* have been described.^56,57^

The atrophy in cases with the *C9orf72* mutation predominates in the frontal region, but also affects the temporal and parietal lobes.^21,36,51^ This atrophy is typically symmetrical, and in some cases there can be minimal atrophy, particularly early in the disease course.^18^ The temporal lobes are generally less affected than in *GRN* or *MAPT* mutations. Some studies have also found atrophy in the thalamus and cerebellum.^36,51^

In terms of neuropathology, the mutation is associated with the presence of TDP-43 positive inclusions similar to those found in FTLD-TDP type B and type A (either type or in combination).^18,58^ FTLD-TDP type A is the one also observed in *GRN* mutations, as previously mentioned, and FTLD-TDP type B is the pathology typically seen in FTD-MND cases, and is characterized by a moderate amount of neuronal cytoplasmic inclusions and sparse dystrophic neurites.^40^ There are also inclusions immunoreactive to antibodies against p62 but negative for TDP-43, particularly in the pyramidal layer of the hippocampus and cerebellum granular cell layer.^58^ These inclusions are immunoreactive to antibodies against dipeptide repeat proteins, such as polyGA, polyGP, and polyGR.^42^ The interactions between the repeat expansion and TDP-43 protein are still unknown, but a study with cell cultures suggested that dipeptide repeat proteins interfere with the ubiquitin-proteasome system, which can affect the homeostasis of the TDP-43 protein.^59^

Recent studies have identified the presence of *C9orf72* hexanucleotide repeat expansions in patients who also had pathogenic mutations in other genes, such as *GRN, MAPT, FUS* and *TARDBP*.^60^ This existence of coexisting mutations is infrequent (less than 2% of cases in a FTD study), but can alter the clinical presentation of the mutations, such as age at onset of symptoms.^60^

***TARDBP* gene.** The *TARDBP* gene is located on chromosome 1p36.22 and encodes TDP-43. TDP-43 is a protein that binds to DNA and RNA, and has multiple functions in RNA metabolism, including regulation of transcription and translation, splicing, nuclear-cytoplasmic transport, and formation of stress granules.^61^

To date, 34 pathogenic mutations have been reported, mainly in cases of familial and sporadic ALS.^15^ Most *TARDBP* mutations are missense, though insertions, deletions and nonsense mutations have been found in some cases.^62^ Cells with cytoplasmic inclusions immunostained with antibodies against TDP-43 typically do not exhibit TDP-43 in the nucleus (where TDP-43 is found in normal cells). This suggests that loss of function in the nucleus and/or toxic effects of the protein in the cytoplasm might underlie neurodegeneration.^61^

The pattern of inheritance is typically autosomal dominant with incomplete penetrance, but individuals with homozygous mutations have been reported.^62^

Mutations in *TARDBP* cause around 3% of familial and 1.5% of sporadic forms of ALS, but are infrequent causes of FTD (with or without MND). Less than 20 probands have been reported in the literature with FTD or dementia.^62,63^ Within the FTD spectrum, FTD-MND, svPPA-MND, bvFTD, CBS and svPPA (including patients with right temporal variant bvFTD and a "bitemporal" bvFTD variant) have been reported.^63,64^ There is great variability in age of onset of symptoms, with reports of onset ranging from between 29 and 77 years.^63^

Mutations were also found in patients with clinical diagnosis of Parkinson's disease, ALS with parkinsonism and ALS with dementia of the Alzheimer type.^65,66^ Atypical presentations were reported, such as bvFTD with chorea and supranuclear gaze palsy, and progressive anarthria.^67,68^

In a patient with svPPA with a *TARDBP* mutation, Gelpi et al. found small intracytoplasmic neuronal inclusions and short neurites immunostained with antibodies against ubiquitin, and TDP-43 (in a pattern similar to FTLD-TDP type A).^69^

***FUS* gene.** The *FUS* gene encodes the fused in sarcoma protein (FUS). This protein belongs to a family of DNA/RNA binding proteins called FET. FUS is involved in various roles related to RNA metabolism, including regulation of transcription, splicing, nuclear-cytoplasmic transport and stress granule formation, besides DNA repair and cellular proliferation.^61,70^

Twenty-three *FUS* mutations have been reported.^15^ Most mutations are missense but insertions and deletions (some causing frameshift mutations) have also been found.^70^ Mutations in this gene seem to cause neurodegeneration both through gain of toxic function, since the FUS mutant protein and overexpression of the normal FUS protein appear to be toxic in animal models, and loss of function mechanisms, because the mutant protein can bind and suppress the functions of normal FUS and of other proteins.^61,70^

Mutations in *FUS* most often present as MND, and are found in about 5% of the familial cases and <1% of sporadic cases of ALS, including juvenile ALS.^62^ Dementia is considered rare in *FUS* mutations and has been reported in only a few families. FTD-MND and ALS-dementia (with or without parkinsonism) have been reported in five families to date,^70,71^ and bvFTD without MND was reported in only two probands (though functional evidence of pathogenicity is still lacking for the mutations in these latter cases).^64,70^

The neuropathological evaluation of ALS cases with *FUS* mutations show cytoplasmic inclusions immunoreactive to antibodies against FUS in neurons and glial cells.^70^

***UBQLN2* gene.** The *UBQLN2* (ubiquilin 2) is located on chromosome Xp11.21.72 Ubiquilin 2 participates in the ubiquitin-proteasome system, comprising part of cellular protein degradation pathways, and mutations in *UBQLN2* appear to affect the function of the ubiquitin-proteasome system.^72^

The inheritance pattern is X-linked dominant, which means that both men and women can be affected.^72^ The penetrance is high, but might be incomplete.^163^

The age of onset is variable, but appears to be significantly lower among men (median of 33 years in men and 44.5 years in women).^72^ Most carriers develop ALS, but some cases with the FTD-MND phenotype have been reported.^72^ Mutations in *UBQLN2* appear to be rare in FTD cohorts, and there is only one FTD patient without DNM reported in the literature.^73^ A kindred with spastic tetraparesis and dementia with behavioral symptoms and a p.P497L mutation was also reported, with symptom onset occurring before 10 years of age in males and between 20-30 years in females.^74^ Only one patient from this family developed ALS.

Studies have shown inclusions immunoreactive to antibodies against ubiquilin 2 in spinal motor neurons and in the hippocampus,^72^ which appear to be co-localized with TDP-43 and FUS.^75^ Ubiquilin 2 positive inclusions are not specific to the cases with mutations in this gene, also being found in cases of ALS and FTD-MND without mutations in *UBQLN2*.^72^

***CHMP2B* gene.** The *CHMP2B* gene (charged multivesicular body protein 2B) is located on chromosome 3p11.2. The CHMP2B protein is part of the ESCRT III complex (endosomal sorting complex required for transport-III), which participates in protein degradation pathways associated with endosomes and lysossomes.^76^

Nonsense mutations in *CHMP2B* are rare genetic causes of FTD, and have been reported in Danish and Belgian families with autosomal dominant patterns of inheritance.^15,76,77^ The most commonly reported phenotype is bvFTD, with a mean age at onset of 68 years and mean disease duration of 10 years.^77^

Missense mutations in the gene have been described in patients with MND (particularly with primary muscle atrophy), FTD-MND, and isolated cases of svPPA and CBS.^76,78^ However, it should be noted that the pathogenicity of these mutations have not been proven unequivocally and most missense mutations have been reported in sporadic cases.

The neuropathological finding in *CHMP2B* mutations is FTLD-U, indicating that this mutation is associated with an as yet unknown proteinopathy.^5^

**Other genes associated with the FTD-MND spectrum.** Mutations in other genes that cause MND were also associated with the FTD-MND phenotype in a few cases or reports of isolated cases.

Mutations in the optineurin gene (*OPTN*) are known to cause open angle glaucoma, and more recently, mutations were identified in cases of ALS and FTD-MND.^79^ Variants in *CHCHD10* (Coiled-Coil-Helix-Coiled-Coil-Helix Domain Containing 10), a gene that encodes a mitochondrial protein, were recently identified in a few families with MND and FTD-MND.^80^

The FTD-MND phenotype was also reported in *SOD1* (superoxide dismutase),^81^ *DCTN1* (dynactin 1),^82^ *ANG* (angiogenin),^83^ *SIGMAR1* (Sigma Non-Opioid Intracellular Receptor 1),^84^ and *DJ-1* (PARK 7) mutations.^85^ Mutations in *SOD1* are the most common genetic cause of ALS, but are rarely associated with cognitive or behavioral symptoms.^81^ Mutations in *DCTN1* are typically associated with Perry syndrome, an autosomal dominant disease characterized by parkinsonism, hypoventilation, weight loss and depression.^82^ Variants in *DCTN1* and in *ANG* are considered risk factors for ALS.^82,83^ Mutations in *DJ-1* cause early-onset Parkinson's disease, with an autosomal recessive pattern of inheritance (PARK 7).^85^ Mutations in *SIGMA1R* were initially described in cases of juvenile ALS.^84^

## GENES ASSOCIATED WITH MULTISYSTEM PROTEINOPATHIES

Mutations in *VCP* were discovered in 2004 were found to cause a complex phenotype, which initially included inclusion body myopathy, Paget disease of the bone and bvFTD and consequently it was commonly referred to by the acronym IBMPFD.^86^ In 2010 however, a study employing exome sequencing found that mutations in *VCP* could also cause familial ALS, thus expanding the phenotypes associated with *VCP* mutations.^87^ Other reports subsequently suggested that these families could occasionally present systemic manifestations such as heart, liver, peripheral nervous system involvement, among others, and therefore the acronym originally used was no longer deemed adequate. Hence, Benatar et al. proposed that this group of phenotypes be renamed "Multisystem proteinopathy" (MSP).^13^

Mutations in *VCP* are the most frequent cause of MSP, but mutations in *HNRNP1* and *HNRNPA2B1* were also recently identified.^88^ Mutations in the *SQSTM1* gene have not been associated with inclusion body myopathy, but have since been found in other phenotypes of MSP.^89^ Other MSP-causing genes will likely be identified in the future, since mutations in these four genes cannot explain all cases of familial MSP.

***VCP* gene.** The *VCP* gene (valosin containing protein) is located on chromosome 9p13.3. The valosin-containing protein is an AAA + protein (ATPase associated with a variety of cellular activities), which participates in multiple cellular processes, such as degradation of ubiquitinated proteins by the ubiquitin-proteasome system and autophagy.^86^

There are currently 18 known missense mutations of the gene.^15^ Mutations in *VCP* seem to affect the protein degradation pathways, which can lead to neurodegeneration.^86^

Transmission occurs in autosomal dominant pattern of inheritance, with variable penetrance depending on the phenotype (around 30% of patients develop bvFTD, 85-90% develop inclusion body myopathy, and 45-50% have Paget's disease).^90^

*VCP* mutations are found in less than 2% of FTD cases and are responsible for 50-75% of cases of familial MSP.^21,89,91^ The onset of symptoms of myopathy (mean age 43 years) and Paget disease (mean age 42 years) usually occur earlier than the onset of FTD symptoms (mean age 55 years).^90^ Mutation carriers can develop all three phenotypes (12%), a combination of two phenotypes (50%) or only one of the phenotypes (38%).^90^ Only 3% develop FTD alone.^90^

The neuropsychiatric manifestations are compatible with bvFTD in most cases, but there are reports of svPPA and dementia of the Alzheimer type.^92^ Disease duration is, on average, 19 years after the onset of myopathy or Paget's disease, but only 6 years, on average, after dementia onset.^92^

Mutations in *VCP* were found in about 2% of familial cases of ALS, and around 9% of individuals with mutations in this gene develop ALS.^87,92^

The neuropathological findings in *VCP* mutations are consistent with FTLD-TDP type D, characterized by frequent short dystrophic neurites, frequent neuronal intranuclear inclusions and sparse neuronal cytoplasmic inclusions.^40^

***HNRNPA2B1* and *HNRNP1* genes.** The *HNRNPA2B1* and *HNRNP1* genes (heterogeneous nuclear ribonucleoprotein A2/B1 and A1, respectively) encode ribonucleoproteins that participate in the RNA metabolism.^13,88^

The mutations described to date are located in the proteins' prion-like domain and are thought to facilitate the proteins' fibrilization and incorporation into stress granules, as well as induce the formation of pathologic cytoplasmic inclusions.^88^

Mutations in these genes are rare causes of familial MSP (0-12%), as well as familial and sporadic ALS.^13,88,91^

There are no reports with neuropathological findings in these mutations, but one study found cytoplasmic inclusions immunoreactive to antibodies against TDP-43, hnRNPA2/B1, and hnRNPA1 in muscle biopsies of patients with a mutation.^88^

***SQSTM1* gene.** The *SQSTM1* gene (sequestosome 1) encodes the p62 protein, which participates in degradation of ubiquitinated proteins by autophagy, and is also involved in cell differentiation, apoptosis and immune responses.^93^

Mutations in *SQSTM1* are missense (predominantly), nonsense or deletions. It is believed that the mutations affect the binding of p62 protein, and therefore, the degradation of proteins.^93^

The most frequently observed phenotype in *SQSTM1* mutations is Paget disease where mutations are found in 25-50% of cases of familial and 5-10% of sporadic cases of Paget disease.^94^ Cases of sporadic and familial ALS, familial bvFTD and familial FTD-MND with *SQSTM1* mutations, have also been identified.^95^ Mutations have been described in families with the bvFTD, Paget disease, and/or ALS phenotypes, but bvFTD and ALS have also been observed in families with no history of Paget disease.^93,95^

Mutations in *SQSTM1* were found in 0.9 to 3% of cases of ALS and FTD (familial and sporadic), and in less than 5% of the familial MSP.^89,93,95^ The onset of symptoms in FTD ranged from between 48 and 73 years.^89,93^ *SQSTM1* mutations have been identified in rare patients diagnosed with nfvPPA, svPPA, PSP and CBS, but the pathogenicity of these mutations has yet to be confirmed.^96^

Inclusions immunoreactive to antibodies against p62, TDP-43 and, in some cases phosphorylated TDP-43 in neurons and glial cells, have been described in patients with mutations in *SQSTM1*.^96^

## FINAL CONSIDERATIONS

It should be noted that despite the important discoveries made to date on the genetics of FTD, there is still much to be discovered, as a significant percentage of familial FTD (around 50-60%) cannot be explained by mutations in the currently known genes.^12,48^ Some of these familial FTD cases without an identified gene might be explained by mutations in genes not typically related to FTD, but that have been reported with the bvFTD phenotype. In fact, mutations in *PSEN1* (presenilin 1) and *PSEN2* (Presenilin 2) that cause autosomal dominant AD, have been found in families with the bvFTD phenotype.^97^ Dementia with frontotemporal features has also been reported in mutations of other genes, such as in *PRNP* (prion protein), that cause genetic prion disease.^98^ The use of next generation sequencing technologies to sequence gene panels, or even whole genomes or exomes, might be useful for identifying pathogenic mutations in familial FTD.

With the identification of key genes associated with FTD, researchers have turned their attention to characterizing the pre-symptomatic stage of the disease in an effort to identify biomarkers that can be used to evaluate response to (future) therapies. Several studies have been conducted with different mutations and have identified mild cognitive symptoms, changes in brain glucose metabolism or structural neuroimaging, and/or changes in functional connectivity in pre-symptomatic mutation carriers.^99^ A recent study suggested structural changes might appear at least ten years (and up to 25 years) before the onset of symptoms.^100^

The ultimate goal of research in pre-symptomatic and symptomatic individuals with monogenic forms of FTD is to develop treatments that modify the course of the disease or even prevent its onset. Among the mutations associated with FTD, the discovery of treatments for *GRN* mutations are the most promising. As *GRN* mutations cause disease through haploinsufficiency, compounds are being investigated to increase gene expression (such as with the use of histone deacetylase inhibitors) or protein secretion. A multicenter phase-two clinical trial with a histone deacetylase inhibitor (FRM-0334) in patients with *GRN* mutations is ongoing (Clinicaltrials.gov NCT02149160).
